# The impacts of climate change on the abundance and distribution of the Spotted Wing Drosophila (*Drosophila suzukii*) in the United States and Canada

**DOI:** 10.7717/peerj.3192

**Published:** 2017-04-06

**Authors:** Aaron B. Langille, Ellen M. Arteca, Jonathan A. Newman

**Affiliations:** 1 School of Environmental Sciences, University of Guelph, Guelph, Ontario, Canada; 2 Department of Mathematics and Computer Science, Laurentian University, Sudbury, Ontario, Canada; 3 Department of Integrative Biology, University of Guelph, Guelph, Ontario, Canada

**Keywords:** *Drosophila suzukii*, CMIP5, Invasive species, Elevated temperatures, Soft-skinned fruit industry, Global circulation model

## Abstract

*D. suzukii* is a relatively recent and destructive pest species to the North American soft-skinned fruit industry. Understanding this species’ potential to shift in abundance and range due to changing climate is an important part of an effective mitigation and management strategy. We parameterized a temperature-driven *D. suzukii* population dynamics model using temperature data derived from several Global Circulation Models (CMIP5) with a range of relative concentration pathway (RCP) predictions. Mean consensus between the models suggest that without adaptation to both higher prolonged temperatures and higher short-term temperature events *D. suzukii* population levels are likely to drop in currently higher-risk regions. The potential drop in population is evident both as time progresses and as the severity of the RCP scenario increases. Some regions, particularly in northern latitudes, may experience increased populations due to milder winter and more developmentally-ideal summer conditions, but many of these regions are not currently known for soft-skinned fruit production and so the effects of this population increase may not have a significant impact.

## Introduction

Climate change is likely to alter the abundance and distribution of invasive species, via changes in: introduction pathways, effectiveness of management strategies, and altered climatic constraints (Hellmann et al., 2008). Changes in key environmental factors such as temperature (Ward & Masters, 2007) will influence species’ ability to survive and thrive in regions for which climate no longer acts as a natural boundary. Invasion and infestation risks may increase as shorter, warmer winters open northern regions to overwintering possibilities and to earlier and longer growth seasons (Bale et al., 2002). Conversely, for some species there may be a reduction in suitability as climate factors begin to exceed mortality and development tolerances. Examining the potential changes in abundance and distribution of pest species due to climate change is vital from economic and food-security perspectives (Ziska et al., 2011).

*Drosophila suzukii* is a relatively recent species-of-concern for soft-skinned fruit producers. While undocumented outside of Japan prior to the 1930s (Kanzawa, 1936), it has increased its geographic range rapidly over the past 35 years and can now be found on every continent except for Antartica (Asplen et al., 2015). *D. suzukii* is particularly concerning because unlike other “vinegar” flies its eggs are deposited preferentially into ripe (or ripening) fruit through the female’s serrated ovipositor (Atallah et al., 2014). Host fruit then spoils due to larval feeding or secondary infection at the puncture site (Goodhue et al., 2011). It is estimated that production losses due to *D. suzukii* infestation could reach 20% of crop yield or above $5 million (USD) from preferred hosts including strawberries, blueberries, cherries, blackberries and raspberries.

As with most insects, temperature is among the critical factors influencing *D. suzukii* population size and consequently infestation potential. Several laboratory studies have highlighted the role of temperature in development and mortality and as such baseline physiological data is available. For example, Ryan et al. (2016) found that mortality thresholds occurred at 5 °C (lower) and 35 °C (upper), that no adult eclosion occurred at temperatures less than 8.1 °C or above 30.9 °C and that optimal temperatures for development and reproductive output occurred at 28.2 °C and 22.9 °C respectively. Similarly, Tochen et al. (2014) found the optimal rate of population growth at 22 °C and development rates occurred at minimal, optimal and maximal temperatures of 13.4 °C, 21.0 °C, and 29.3 °C respectively.

**Figure 1:**
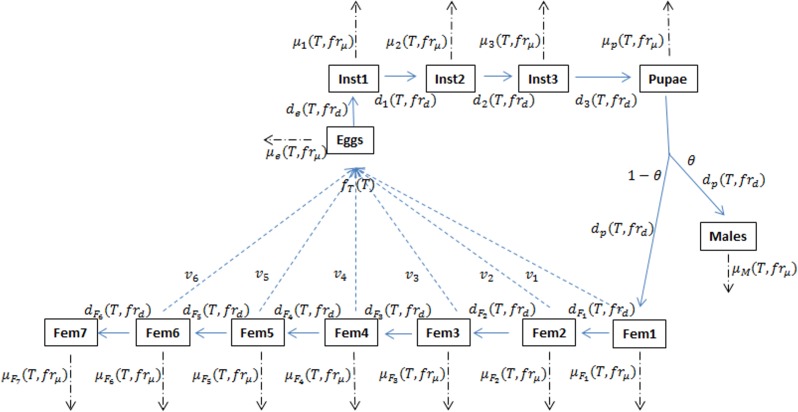
Schematic of the *D. suzukii* population dynamics model presented in Langille et al. (2016). This schematic highlights population stage structure and relationships between state variable equations including principal input and output parameters. Fecundity (*f*), development (*d*) and mortality (*μ*) processes are temperature (*T*) dependent while development and mortality also rely on the fruit quality sub model (*fr*). In addition to eggs, pupa and adult males, the model contains three juvenile instar stages and seven adult female stages in order to account for variation in egg viability (*v*). θ represents the ratio of males to females and is typically set at 0.5. Solid arrows indicate developmental stage transition, dashed arrows indicate fecundity and dot-dashed arrows indicate mortality.

Langille et al. (2016) have developed a mechanistic model of *D. suzukii* population dynamics (Fig. 1, see also Gutierrez, Ponti & Dalton, 2016), including submodel considerations for reproductive diapause and influence of fruit quality or viability. The principle environmental driver for the model is temperature and key biological mechanisms such as mortality, fecundity and development rates (for each life stage) have been parameterized wherever possible using the laboratory data of Ryan et al. (2016) and other literature-based estimates (Emiljanowicz et al., 2014). The model does not currently account for overwintering so all simulations run for a single year and typically begin with an initial number of adult females introduced on the date when diapause would be terminated due to adequately warm temperatures. Parameterizing the model with observed temperatures from 1993 to 2013, Langille et al. demonstrated potential relative population levels for twelve berry producing regions across Canada and the United States. The goal of that work was to demonstrate that, assuming temperature is a key determinant of population size, not all fruit-producing regions are at equal risk for infestation potential or severity, despite the almost ubiquitous presence of *D. suzukii* across North America. Our goal in the present work is to explore how infestation-potential may be affected due to projected climatic change.

## Materials and Methods

Projecting future climate on a global scale is an exceptionally complex task given the number of processes and interactions that occur in the biotic and abiotic environment. Despite the challenges, Global Circulation Models (GCMs) continue to be refined and offer projected climate based on various modelled assumptions about those processes and interactions. The most recent suite of models follow the Coupled Model Inter Comparison Project Phase 5 (CMIP5) framework and differ from their predecessors, in part, in their use of Representative Concentration Pathways (RCPs). Each of the RCPs represents a potential future trajectory of the principle forcing agents (measured in W/m^2^) driving climatic change. In simplified terms RCP2.6 represents a “peak-and-decline” scenario where radiative forces peak by mid century and decline by 2100. Both RCP4.5 and RCP6.0 are scenarios where stabilization of radiative forces occur by 2100, while in RCP8.5, these forces continue to increase beyond 2100 (Van Vuuren et al., 2011). A subset of possible temperature projections was downloaded from the archives of the Downscaled CMIP3 and CMIP5 Climate and Hydrology Projections (ftp://gdo-dcp.ucllnl.org/pub/dcp/archive/cmip5/bcca). Specific climate models were chosen where projections were available for each of the four Representative Concentration Pathways (RCPs) 2.6, 4.5, 6.0, 8.5. Selected climate models included the BCC-CSM1.1, CCSM4, GFDL-EMS2G and ISPL-CM5A-LR (Table 1), all of which conform to the Coupled Model Intercomparison Project Phase 5 (CMIP5) framework.

**Table 1:** Official contributor name, institute ID and model name for the GCMs used herein.

Modelling center or group	Institute ID	Model name
Beijing Climate Center, China Meteorological Administration	BCC	BCC-CSM1.1
National Center for Atmospheric Research	NCAR	CCSM4
NOAA Geophysical Fluid Dynamics Laboratory	NOAA GFDL	GFDL-EMS2G
Institut Pierre-Simon Laplace	ISPL	ISPL-CM5A-LR

The temperatures obtained were derived from projections downscaled via Bias Corrected Constructed Analogs (BCCAv2, Maurer et al., 2007; Brekke et al., 2013) and consist of daily minima and maxima at 1 degree (latitude/longitude) spatial resolution. We computed daily mean as (minimum + maximum)/2. Data were averaged over thirty-year time spans such that three time periods 2020s (2010–2039), 2050s (2040–2069) and 2080s (2070–2099) were produced. Each simulation began with 10 fecund females introduced on the date for which temperature would break reproductive diapause (18 °C). The fruit development index which controls the rate at which fruit reaches maturity was set to 4.0 and fruit was available at maximum quality for 50 days before beginning to decline. Although multiple harvests per year were not included in these simulations, the model does account for the availability of wild hosts (Lee et al., 2015) by ensuring that the fruit quality sub model continues to have a positive, albeit minimized, effect on development post-harvest (*cf.* Langille et al., 2016). Reproductive diapause is initiated when the simulated grid cell reached 10 daylight hours per day (latitude-dependent). Table 2 provides a summary of the key model parameters and values.

**Table 2:** Key base model parameters for simulations, *cf*. Langille et al. (2016, supplemental) for complete parameter value listing.

Description	Value
Initial population of fecund females (individuals)	10
Diapause termination temperature (°C)	18
Diapause induction date based on daylight hours	10
Fruit development index (*ω*)	4.0
Fruit harvest lag (*h*_*Fr*_, days)	50

## Results

**Figure 2:**
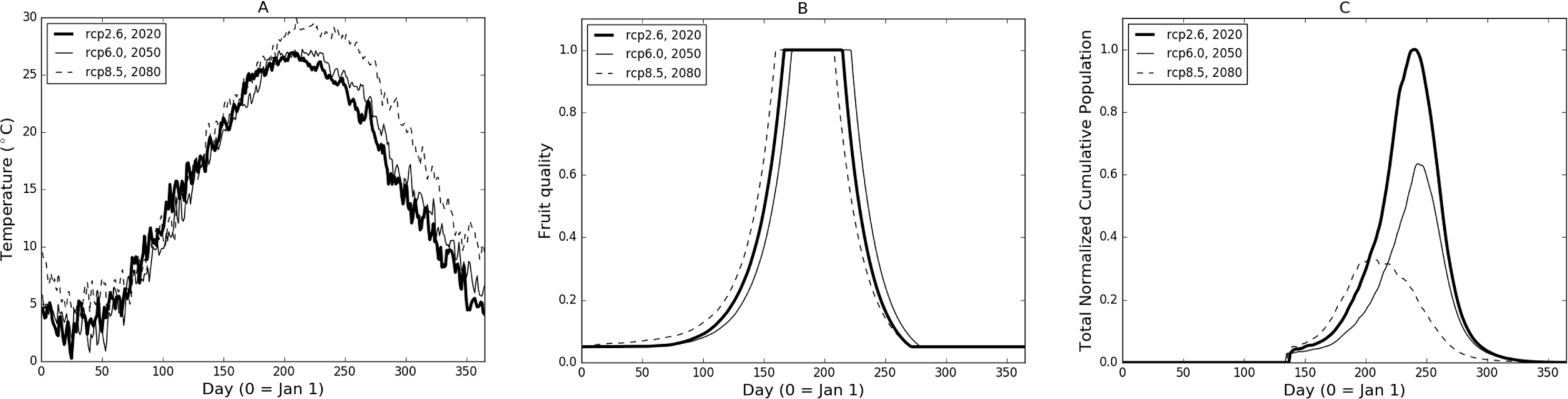
(A) Sample temperature profiles for Burlington, New Jersey from the CCSM4 model with different RCP-date combinations. Model-RCP combinations were selected to illustrate a range of output possibilities. (B) and (C) illustrate fruit quality submodel response and model population based on temperature profiles in (A).

In Fig. 2 we illustrate the base model properties under a subset of temperature scenarios from the CCSM4 model for Burlington, New Jersey. The temperatures for three different RCPs and time frame combinations (Fig. 2A) show a relatively low (RCP2.6-2020), mid (RCP6.0-2050) and high (RCP8.5-2080) range of potential conditions. The difference in the temperature profiles result in varying fruit readiness date (Fig. 2B) and population levels (Fig. 2C). In particular, RCP8.5-2080 produces the earliest optimal fruit in part due to warmer temperatures early in the year while fruit ripens more quickly in RCP2.6-2020 than in RCP6.0-2050, due to slightly elevated temperatures in the March through May range. It is worth noting that the RCP6.0-2050 temperatures are generally higher than those of RCP2.6-2020 in the later part of the year (June and onward) but this occurs after fruit has matured and been harvested. Despite some variation between the curves, there are only 14 days difference between the earliest and latest readiness dates.

In terms of population (Fig. 2C), RCP8.5-2080 initially grows at the steepest rate but peaks relatively early in the year and begins to decline due to temperatures (Fig. 2A) that are both too high to maximize development and contribute to mortality. RCP6.0-2050 growth is slowest due to the lower early-year temperatures which also causes slower fruit quality increases (Fig. 2B). In comparison, RCP2.6-2020 provides maximum growth potential with early warmth and lower mid-to-late year temperatures that continue to foster growth without severely impacting mortality. Considering both reproductive output and development, modelled population growth occurs optimally at temperatures around 24 °C. Quantitatively, temperature profiles for RCP2.6-2020, RCP6.0-2050 and RCP8.5-2080 produce 74, 62 and 47 days respectively where temperatures are ±2 °C from model optimal.

**Figure 3:**
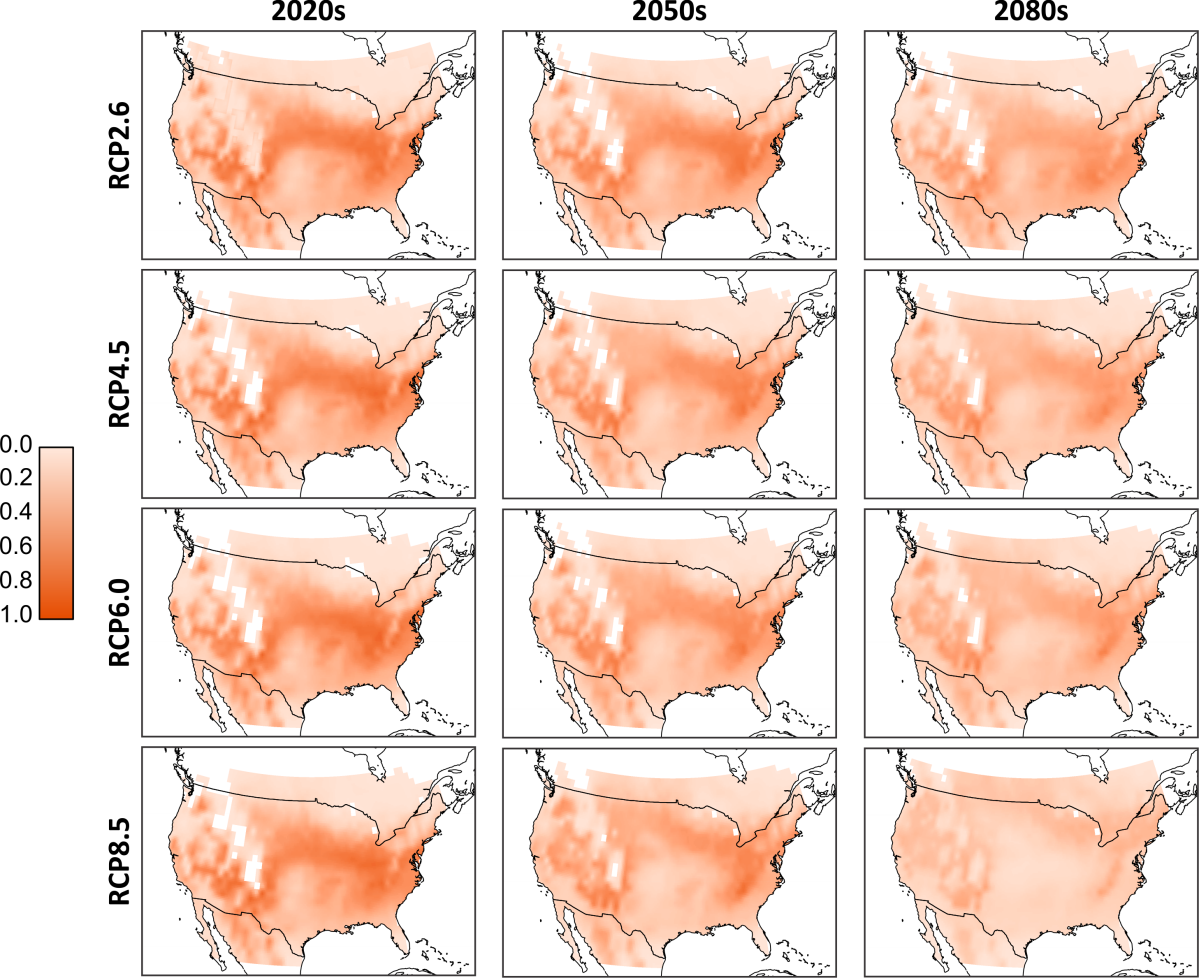
Aggregated consensus maps of the modelled populations. Data are the computed mean normalized total population (all life stages) from all four climate models. Normalization is based on the entire pre-mean data set. White cells located in the interior parts of the maps reflect missing values in the downscaled climate model outputs and a 0.25 degree bi-linear interpolation was applied to all data cells.

As we cannot know *a priori* which of the climate models will produce the most accurate temperature projections, we combined the results to form ‘consensus maps’ (Fig. 3). Simulated population totals (all life stages) were normalized across the entire data set, and means were generated per corresponding RCP and timeframe (i.e., these are averages of the results of the *D. suzukii* model, run for the different climate models). From these consensus maps, we see that the 2020s have the potential for the most extensive high-population distribution across southern Canada and much of the continental United States. Northern Canada and regions occupied by the Rocky Mountains have relatively low populations due to lower temperatures that discourage development and encourage mortality. Similarly, the southern extent of the United States has relatively low populations due to excessively high temperatures. There is a noticeable shift in population distribution moving to the 2050s as the central US becomes warmer and populations decline. Some northern central regions in Canada increase in population as temperatures become more favourable. These trends continue into the 2080s as the central and northern US become too warm, central Canada becomes warmer and the most favourable conditions in North America are located primarily to the north-eastern and western US as well as a few regions in southern Canada. While there is little qualitative difference between the model output in the RCP scenarios for the 2020 temperature projections, by the 2080s there is an evident decline in relative population levels in RCP 8.5.

As with the climate model, it is difficult to predict which RCP scenario will produce the most likely greenhouse gas emissions, so we further aggregate the model results by folding the model-RCP combinations into per-time frame consensus maps. Similar to Figs.  3, in 4A we see a general reduction in population sizes with the exception of moderate increases in the far northern regions as we move into later time frames. While these mean populations give an overall impression of possible trends it is important to acknowledge the difference in values produced by the various model-RCP combinations.

**Figure 4:**
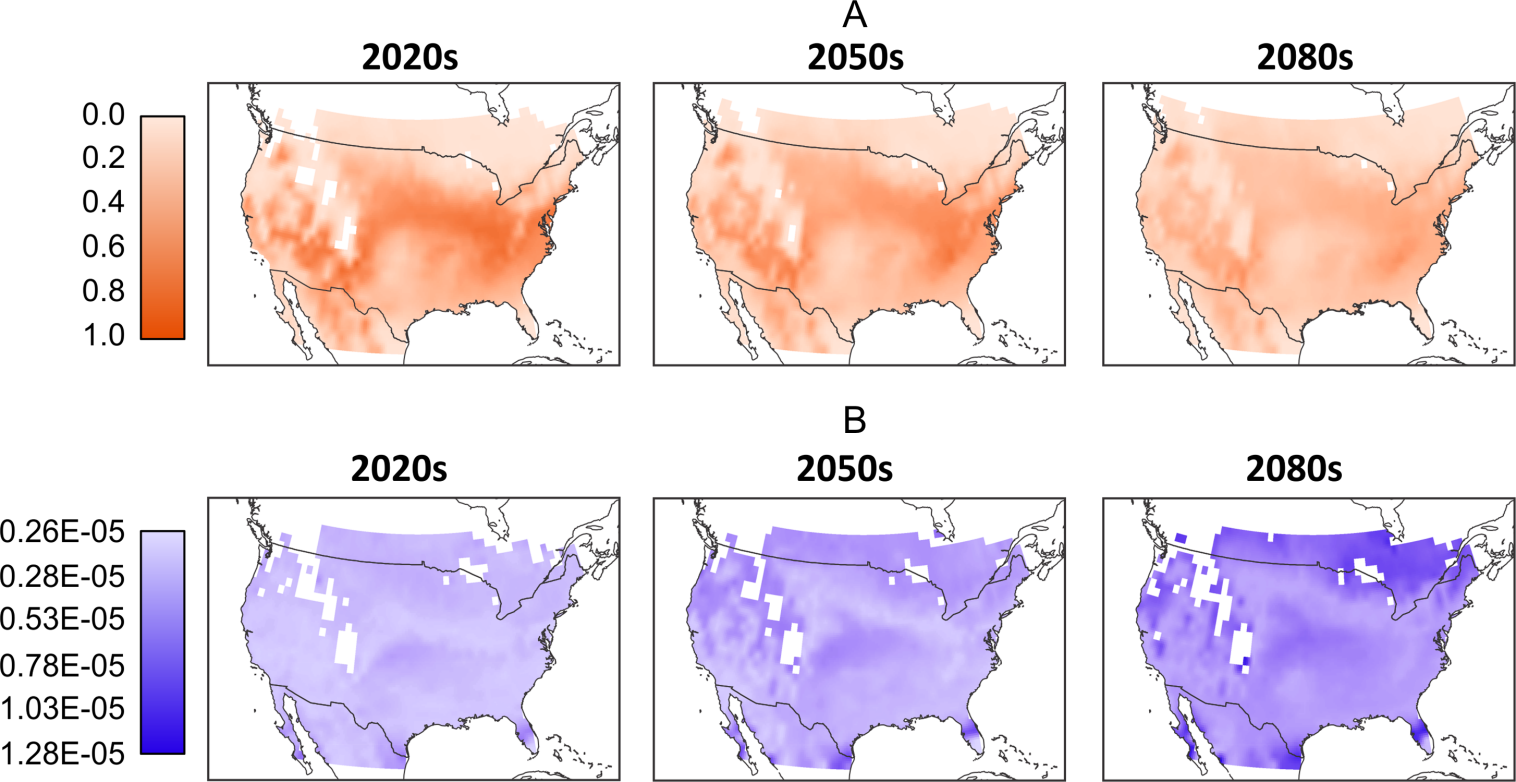
(A) Mean normalized population distribution across North America per-timeframe. Normalization occurs across the entire data set and mean is across all models and RCPs. (B) Corresponding per-timeframe coefficient of variation across all models and RCPs. Coefficient of variation is based on the normalized population data. White cells located in the interior parts of the maps reflect missing values in the downscaled climate model outputs and a 0.25 degree bi-linear interpolation was applied to all data cells.

In Fig. 4B we illustrate these differences by showing the coefficient of variation between the normalized population values. For the 2020 model-RCP combinations we see relatively low values across the map. The exceptions are larger values to the extreme south in Florida, California and Texas. The 2050s show a larger range and an increase in high-variation locations, particularly in the extreme south and north. These trends continue into the 2080s, where we note larger variations over larger areas to the north south and west. Despite these large areas of variation, there remain areas of moderate and even low variation particularly in the interior.

**Figure 5:**
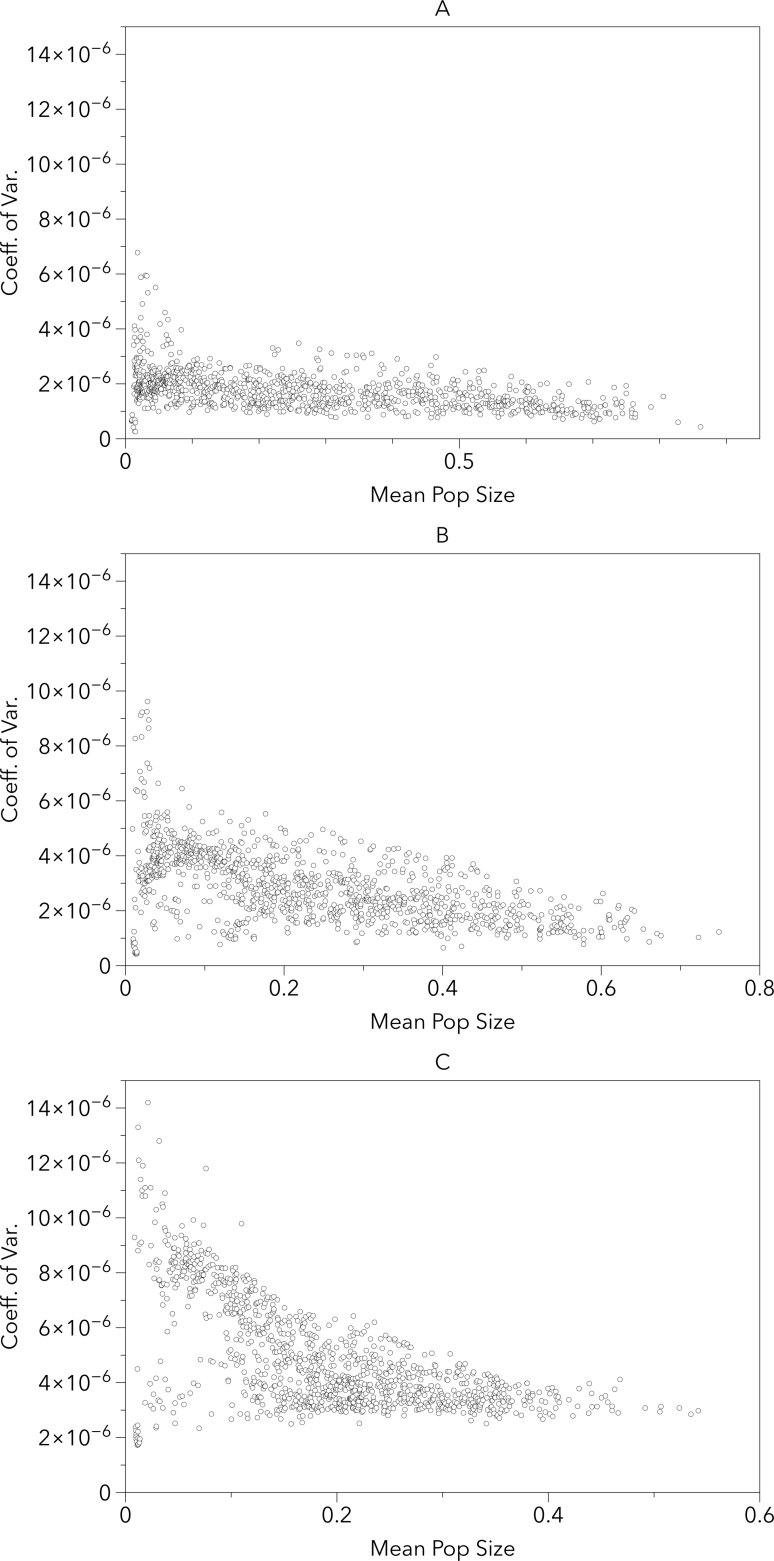
Grid cell coefficient of variation in population sizes versus the grid cell mean population size for the 2020s, 2050s and 2080s (A, B, C respectively). The uncertainty in the population sizes that result from the differences between the climate model–RCP combinations tends to be larger as the mean of those population sizes gets smaller. Not surprisingly, the uncertainty is also larger later in the century than  earlier.

Figure 5 provides an alternate illustration of the overall uncertainty (i.e., larger coefficients of variation) among the model-RCP combinations. It shows both a negative correlation between coefficient of variation and mean population size and an increase in variation as the timeframe increases. This suggests that the largest per-cell uncertainty among model-RCP temperature estimates are in regions that would typically produce smaller populations (too warm or too cool) and that these estimates continue to diverge as the timeframe increases.

**Figure 6:**
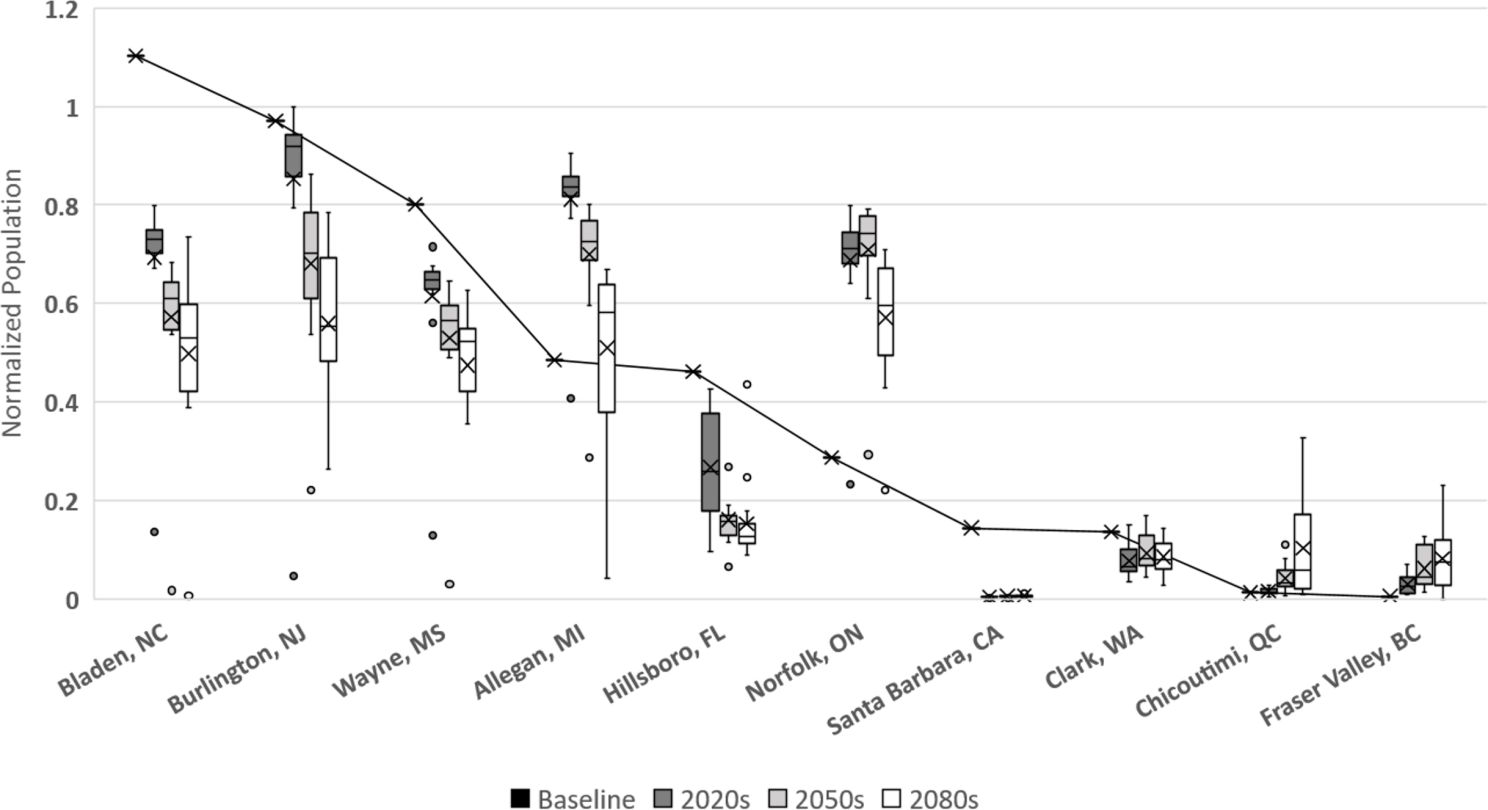
Normalized populations for historic temperatures and GCM projections. GCM projections are averaged over all climate model-RCP combinations where × denotes mean population, whiskers denote maximum and minimum values, interior bar marks quartiles and small circles denote outliers. For averaged historic temperatures, large circles represent population normalized to GCM results.

In Langille et al. (2016) we presented potential modeled populations for some of the more economically important berry producing counties and townships in the United States and Canada. Observed mean daily temperatures for a 20-year span (1993–2013) were obtained via the National Climatic Data Center (http://www.ncdc.noaa.gov/) for US locations and through Environment Canada (http://climate.weather.gc.ca/) for Canadian locations. For each location mean daily temperatures across the timespan were calculated. Figure 6 illustrates how the GCM model and RCP scenarios may affect population simulations in comparison with these historic temperatures. More than half of the locations (including: Bladen, North Carolina; Burlington, New Jersey; Wayne, Mississippi; Hillsborough, Florida; Santa Barbara, California; and Clark, Washington) have historic populations higher than all projected averages. This suggests that these areas are currently in or near the time frame for maximum population growth potential and that as temperatures increase over time populations should decrease. For the remaining locations (including Allegan, Michigan; Norfolk, Ontario; Chicoutimi, Quebec; and Fraser Valley, British Columbia) the historic population is consistently lower than all of the projected averages suggesting that growth potential has yet to be maximized in those locations. It is also worth noting that most of the populations are projected to decrease by the end of the present century, including Allegan and Norfolk. The exception to this decline are the two northern most regions (Fraser Valley and Chicoutimi) which may continue to increase though their population levels remain relatively small.

## Discussion

Understanding how pests such as *D. suzukii* may shift in range and density over time is an important part of an effective mitigation and management strategy (Asplen et al., 2015; Dreves, 2011). By combining various climate models and emissions scenarios we provide a mean outlook as to how increasing temperatures may affect infestation potential over the coming decades. The results of our simulations offer a somewhat mixed forecast for *D. suzukii* infestations. They suggest that some regions in North America may already be at or near the peak of their population growth potential. Distributions and infestation hot-spots may shift over the next two or three decades but as we move closer to the end of the century, depending on the degree of climatic change, we may see a marked decline in population growth potential from this destructive pest as temperatures become too warm to sustain development and mortality increases. Beyond sustained higher temperatures, an increase in short-term heat events in susceptible regions may adversely affect crop output (Teixeira et al., 2013) and subsequently lower pest population potential. In contrast, some northern regions may become more favourable for *D. suzukii* population growth and although many of these regions are not currently known for commercial soft-skinned fruit production currently this too may change over time (Olesen & Bindi, 2002).

It is important to address the simplifications of the model used in this work. At this time there is no consideration for overwintering and as such the model is limited to single year simulations. Although empirical work continues to highlight the biological mechanisms and consequences of overwintering (Wallingford, Lee & Loeb, 2016; Wallingford & Loeb, 2016; Shearer et al., 2016; Stephens et al., 2015) it has not yet been included in the model (see discussion in Langille et al., 2016). As a result, these simulations do not account for inter-annual population differences due to winter survival rates and subsequently presume that all regions begin with the same starting population size each year, which does not specifically reflect migrations (immigration or emigration) due to environmental or anthropogenic factors. Also, considerations for other mortality and development influencing factors such as humidity are not included but are likely to impact population growth (Tochen et al., 2016) while others such as predation and (parasitism) have been greatly simplified to reduce complexity.

Perhaps the most important limitation of the current work is the lack of accounting for evolutionary adaptation that may well occur over the course of the century. While this simplification may be reasonable when considering both the 2020s and historic temperatures, it may be more reasonable to assume that some adaptive changes are likely to occur before the 2050s and 2080s. Both Umina et al. (2005) and Gilchrist et al. (2004) have demonstrated that rapid climate-driven physiological and adaptive change is possible in other species (*D. melanogaster* and *D. subobscura* respectively) although Kimura (2004) found no difference in thermal tolerance between cool and warm latitudinal strains of *D. suzukii* in its native region of Japan. Previous work has also shown that Drosophila species (such as *D. melanogaster*) can be cold and heat-hardened if short term temperature changes are non-lethal and that over longer term exposures acclimation can occur (Wallingford, Lee & Loeb, 2016; Hoffmann, Sørensen & Loeschcke, 2003). Heat hardening may also increase desiccation resistance (Hoffmann, 1990). What remains unclear at this time are the exact thresholds for which evolutionary adaptations will protect species such as *D. suzukii* from temperature and humidity extremes and the developmental and reproductive trade-offs that may occur from such adaptations.

**Figure 7:**
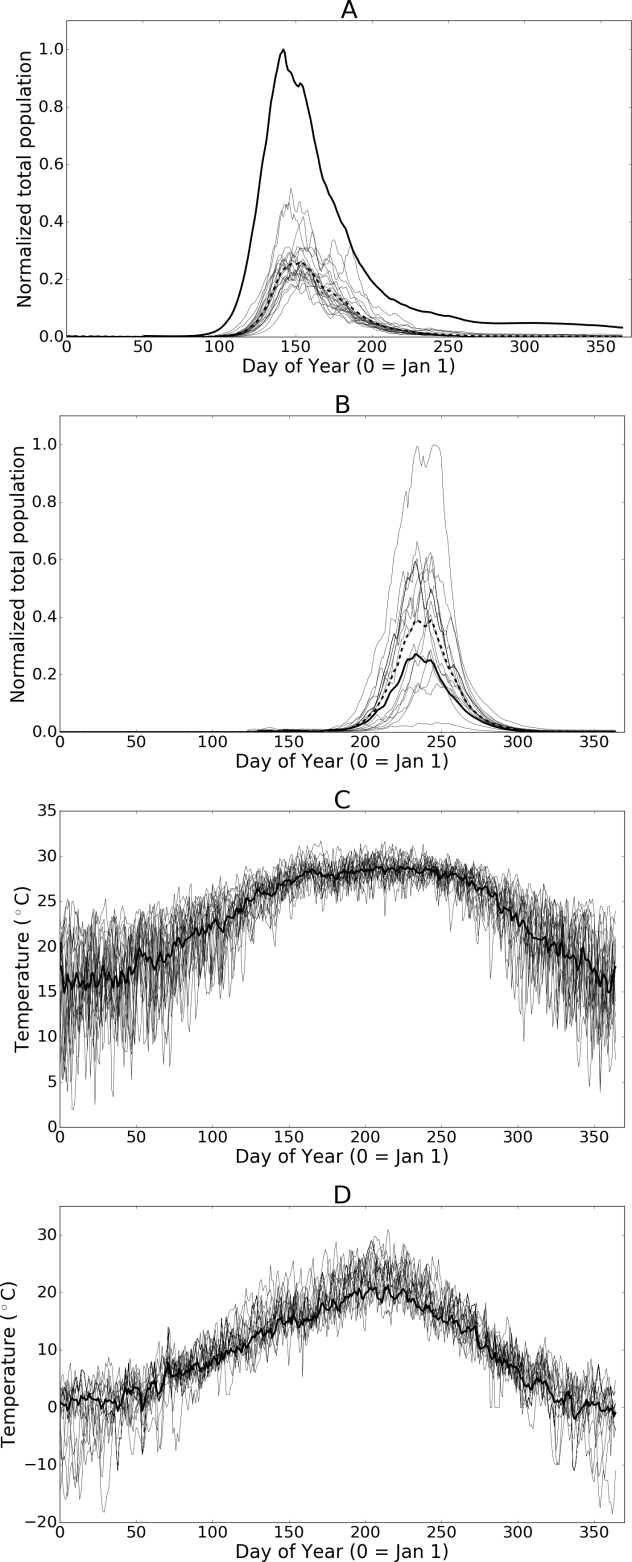
(A) and (B) Normalized populations over 20 years of historic data (1993 through 2013) for Hillsborough, Florida and Fraser Valley, British Columbia respectively. The dashed line indicates the mean annual population while the bold-solid line indicates the population for the mean temperatures across all years. (C) and (D) illustrate the daily temperature data for all years and the mean daily temperatures across all years for Hillsborough, Florida and Fraser Valley, British Columbia respectively.

Although it is standard practice to average temperatures over larger time spans when using climate model data, the averaged data dampens short-term drops or spikes in temperature that might adversely affect population sizes. As such, the simulation results presented herein offer insight into the average potential for growth but do not highlight the extent of inter-annual variation that may occur. To illustrate the effect of using mean temperature data with this model in Figs. 7A and 7B we show a set of population curves for the historic temperature profiles (annual temperatures from 1993 to 2013). We also show the average of the population curves and the population when the simulation is run using the average temperatures over the 20-year time period. In some cases, such as with Hillsborough Florida, averaging the temperatures creates a near-optimal profile by greatly reducing the number of days where temperatures are either too high or too low to support development. The resulting population is larger than any of the individual observed temperature profiles would otherwise suggest. Conversely averaging the temperatures at Fraser Valley, British Columbia, results in a profile where most days are well below optimal growth temperatures. The individual historic temperature profiles, while more variable than the mean, offer the population more opportunity for growth. The resulting population from the averaged temperature profile is significantly smaller than most of the individual profiles.

Given the inherent uncertainties in both the base population dynamics model and the GCM-derived temperature data, the work presented here is not intended to be quantitative predictions. Rather we hope that these results, when considered with comparable modelling efforts on *D. suzukii* or similar pest species, will help to guide and refine empirical and theoretical work on the mechanisms that drive population levels and highlight the range of possible outcomes as we enter a critical time of rapid climatic change. As models continue to highlight our level of understanding of population-driving processes, empirical studies will continue to provide new and increasingly accurate quantitative inputs. Similarly, as GCMs continue to develop and our understanding of complex global environmental processes matures, opportunities for explorative and potentially predictive modelling of invasive species will also improve.

##  Supplemental Information

10.7717/peerj.3192/supp-1 Supplemental Information 1 Normalized historic and GCM populations Raw data for normalized populations of historic temperatures and GCM projections as shown in Fig. 6. GCM projections are averaged over all climate model-RCP.

10.7717/peerj.3192/supp-2 Supplemental Information 2 Individual raw data maps Raw data used to create consensus maps (shown in Figs. 3 and 4A) coefficient of variance maps (shown in Fig. 4B) and in scatter plots shown in Fig. 5.

10.7717/peerj.3192/supp-3 Supplemental Information 3 Population and fruit quality index for base model Raw data used to illustrate fruit quality submodel (shown in Fig. 2B) and resulting populations derived from the base model for sample GCM scenario and RCP combinations (shown in Fig. 2C).

10.7717/peerj.3192/supp-4 Supplemental Information 4 Populations per year and for mean annual temperature Raw data used to illustrate difference between mean populations and populations derived from mean temperatures (shown in Figs. 7A and 7B). Temperature data is also included (shown in Figs. 7C and 7D).

## Additional Information and Declarations

### Data Availability

The following information was supplied regarding data availability: The raw data has been supplied as a Supplementary File.
